# A unified framework for the measurement of mobility in older persons

**DOI:** 10.1093/ageing/afad125

**Published:** 2023-10-30

**Authors:** Marla K Beauchamp, Qiukui Hao, Ayse Kuspinar, Jotheeswaran Amuthavalli Thiyagarajan, Christopher Mikton, Theresa Diaz, Parminder Raina

**Affiliations:** School of Rehabilitation Science, McMaster University, Hamilton, ON, Canada; McMaster Institute for Research on Aging, McMaster University, Hamilton, ON, Canada; School of Rehabilitation Science, McMaster University, Hamilton, ON, Canada; School of Rehabilitation Science, McMaster University, Hamilton, ON, Canada; McMaster Institute for Research on Aging, McMaster University, Hamilton, ON, Canada; Ageing and Health Unit, Department of Maternal, Newborn, Child and Adolescent Health and Ageing, WHO HQ, Geneva, Switzerland; Demographic Change and Healthy Aging Unit, Social Determinants of Health, World Health Organization, Geneva, Switzerland; Epidemiology, Monitoring and Evaluation Unit, Department of Maternal, Newborn, Child and Adolescent Health and Ageing, WHO HQ, Geneva, Switzerland; McMaster Institute for Research on Aging, McMaster University, Hamilton, ON, Canada; Department of Health Research Methods, Evidence, and Impact, McMaster University, Hamilton, ON, Canada; Labarge Centre for Mobility in Aging, McMaster University, Hamilton, ON, Canada

**Keywords:** older adults, physical function, functional status, mobility assessment, psychometrics

## Abstract

Mobility is often referred to as a ‘sixth vital sign’ because of its ability to predict critical health outcomes in later adulthood. In the World Health Organization (WHO) World Report on Aging and Health, mobility is described as movement in all its forms whether powered by the body or a vehicle. As such, mobility encompasses basic physical actions such as getting up from a chair and walking, as well as activities such as exercising, driving and using public transportation. A plethora of measurement tools have been developed to assess various aspects of mobility; however, there is wide variability in the mobility constructs being measured which limits standardisation and meaningful comparison across studies. In this paper, we propose a comprehensive framework for measuring mobility that considers three distinct facets of mobility: perceived mobility ability (‘what can you do’), actual mobility ability (‘what you actually do’) and locomotor capacity for mobility (‘what could you do’). These three facets of mobility are rooted in the three components of healthy aging endorsed by the WHO: functional ability, intrinsic capacity and environments. By proposing a unified framework for measuring mobility based on theory and empirical evidence, we can advance the science of monitoring and managing mobility to ensure functional ability in older age.

## Key Points

- Mobility is a strong predictor of adverse health outcomes and is viewed as an important component of healthy aging.
- We propose a unified framework for measuring mobility consisting of three facets: perceived mobility, actual mobility and locomotor capacity for mobility.
- The proposed framework will help advance the science and discourse of mobility measurement for healthy aging.

## Introduction

Mobility is a strong predictor of health outcomes in later life and is recognised as a critical component of healthy aging. The World Health Organization’s (WHO) World Report on Aging and Health describes mobility as movement in all its forms, whether powered by the body (with or without an assistive device) or a vehicle [1]. Mobility therefore includes simple movements such as getting up from a chair as well as more complex tasks such as walking, exercising and driving a car [1, 2]. Numerous outcome measures have been developed to assess mobility in population and clinical settings. Some reflect self-perceptions of mobility difficulty (e.g. physical function scales), while others capture the frequency of mobility activities undertaken in daily life (e.g. physical activity and life-space mobility measures). Furthermore, many performance-based measures of capacity (e.g. gait speed) are also commonly used as indicators of mobility. The variability in the mobility constructs being measured and lack of standardisation in terminology makes meaningful comparison across studies difficult. In this issue of Age and Aging, three systematic reviews related to mobility measurement were commissioned by the WHO. Each review focuses on different yet related aspects of mobility important for monitoring healthy aging. To distinguish between them and to advance scientific discourse, in this paper, we propose a unified framework for measuring mobility in older persons.

Previous frameworks, such as Webber’s mobility framework or the International Classification of Functioning Disability and Health, focus on broad determinants of mobility and functioning involving both environmental and personal factors [3, 4]. Although useful in understanding the associations between mobility and related outcomes, these frameworks provide little guidance in terms of how mobility should be measured, especially from the individual perspective.

Traditional discourse around mobility measurement has focused on two main categories of outcome measures: self-report measures that reflect an individual’s perspective of their mobility and performance-based measures that reflect a rater’s evaluation of an individual’s performance on specific physical tests. Many studies have debated and compared the two approaches [5–8] with most concluding that they represent complimentary but different mobility constructs due to the demonstrated differences in their underlying factors, predictive validity, and responsiveness, that vary across studies. Importantly, this literature is difficult to interpret as most studies compared measures that assess different mobility tasks (e.g. walking vs. daily activities) as well as different aspects of mobility (i.e. self-reported ability to complete a task vs. frequency of task performance). These conceptual differences are particularly relevant when considering self-report measures, which include questions that can be framed in different ways. For example, we have previously shown that, compared to measures that ask older adults ‘how limited are you…’, those that ask about frequency of task performance (‘how often do you…’) have more predictive validity for adverse outcomes, yet are less amenable to change [9].

Despite extensive literature showing differences in psychometric properties among various mobility outcome measures, little attention has been paid to distinguishing different aspects of mobility measurement beyond the categories of self-report and performance-based measure. Twenty-five years ago, Thomas Glass proposed three ‘tenses’ for the measurement of functioning in older adults: hypothetical, experimental and enacted [10], that we suggest still has much relevance today. In Glass’s model, the hypothetical tense of function refers to a person’s perceived function; the experimental tense of function refers to a person’s functional capability assessed in a laboratory; and finally, enacted function refers to a person’s actual performance at home. The 1998 paper further presents empirical evidence showing a consistent level of discordance between what people say they can do and what people actually do at home, emphasising the need to consider multiple aspects of function to ascertain an older person’s true functional ability.

### Proposed framework

**Figure 1:**
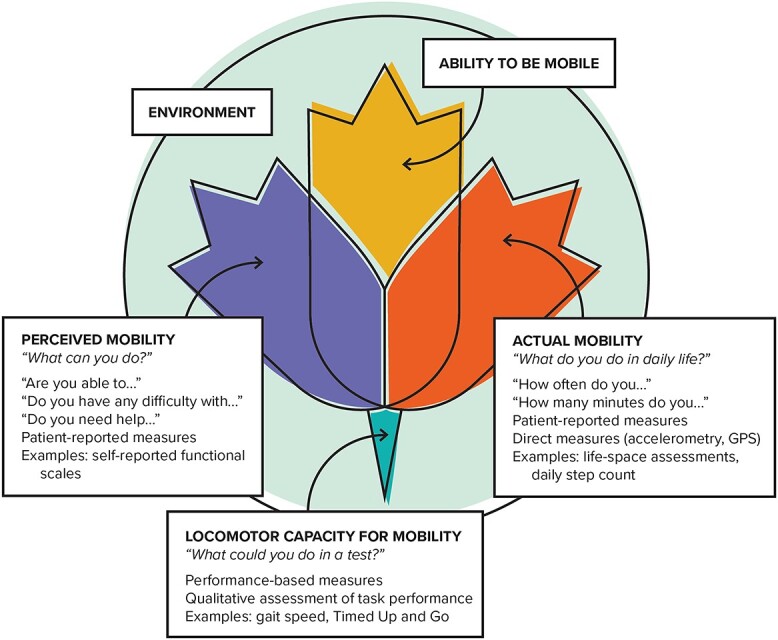
Unified framework for mobility measurement in older persons.

The WHO describes healthy aging as ‘the process of developing and maintaining the functional ability that enables well-being in older age’ and further qualifies that functional ability is a product of the intrinsic capacity of the individual and the environment within which a person lives and interacts [1]. Functional ability, intrinsic capacity and the environment thus make up the three components of healthy aging endorsed by the WHO. Within functional ability, the ‘ability to be mobile’ is specified as one of five critical domains. Building on the original Glass model of functioning [10] and the WHO’s conceptualisation of healthy aging and mobility [2], we propose a unified framework for mobility measurement in older populations (see Figure 1).

Our framework outlines three facets for comprehensive mobility measurement in older adults: (i) perceived mobility, (ii) actual mobility and (iii) locomotor capacity for mobility that are grounded in the WHO healthy aging model. *Perceived mobility* answers the question ‘What can you do?’ (i.e. Glass’s hypothetical tense) and refers to self-report measures that ask about ability or difficulty with mobility task performance. *Actual mobility* answers the question ‘What do you do in daily life?’ (i.e. Glass’s enacted tense) and can include both self-report measures of frequency and duration of activities done in the home and community setting, as well as direct measures of ‘free-living’ mobility obtained via accelerometry or GPS devices. Together, *perceived mobility* and *actual mobility* make up the *ability to be mobile* domain of functional ability—the first major WHO component of healthy aging. Finally, *locomotor capacity for mobility* refers to ‘What could you do in a test?’ (i.e. Glass’s experimental tense) and is typically assessed by performance-based measures of mobility such as tests of gait speed or balance. This conceptualisation is in line with the ‘locomotor capacity’ domain [11] of intrinsic capacity—the second major WHO component of healthy aging, representing the core physical and mental capacities of an individual.

Of note, as a sub-domain of intrinsic capacity, locomotor capacity is broadly described as an individual’s physical capacity to move and includes functions of the joints, bones, reflexes and muscle strength [11]. Within our conceptualisation, measures of locomotor capacity *for mobility* would encompass only those measures that directly align with the concept of mobility versus all the potential measures that might be included as assessments of locomotor capacity (e.g. tests of joint function would be excluded). Another consideration is that in our framework, all three facets of mobility are grounded in the context of a person’s environment, which includes the home, community and broader society. In the original WHO conceptualisation, intrinsic capacity is independent of environment. However, given the many potential environmental features that can influence a person’s physical performance on a test (e.g. straight vs. curved walking path, air quality, etc.), we contend that locomotor capacity for mobility is not independent of the environment. As such, a person’s overall *ability to mobile* relies upon their underlying locomotor capacity for mobility and their environment as shown in Figure 1.

## Discussion

Our framework extends previous conceptualisations of measurement of late-life functioning and is also consistent with the definitions and terminology endorsed by the WHO. The distinction between the three mobility facets can be easily understood by examining the overarching questions for each facet as shown in Figure 1. A standardised language for mobility measurement has important applications for gerontological research and practice, in which mobility is often considered a ‘6^th^ vital sign’ because of its ability to predict critical health outcomes.

In this WHO special issue of Age and Aging on measurements of healthy aging, three systematic reviews are included on the psychometric properties of mobility-related measures that illustrate the usefulness of our framework for evidence synthesis. The first review focuses on *perceived mobility* [12] and includes self-report measures of older peoples’ perception of their ability to be mobile (what they say they can do). Examples include scales that ask, ‘Are you able to…?’, ‘Do you have any difficulty…?’, ‘Do you require assistance …?’ and so on. Most patient-reported mobility measures fall in this category. The second systematic review focuses on *actual mobility* [13] measurement through life-space mobility assessments that consider the frequency and extent of one’s mobility both in the home and in the community. Furthermore, there is a third systematic review on locomotor capacity that includes relevant performance-based measures of mobility such as gait speed and Timed Up and Go, consistent with our framework’s delineation of *locomotor capacity for mobility* [14].

We acknowledge several potential limitations of our framework. Mobility largely depends on one’s values and perspectives, and is closely related to cognitive, psychosocial and environmental factors. Local infrastructure and physical, natural, social and cultural domains of one’s environment can be enabling or disabling (e.g. access to a vehicle or mobility device can impact one’s ability to be mobile). Our framework is based on an individual perspective that may not apply to studies with organisational perspectives. It also does not delineate if there is a hierarchy among the mobility facets or consider the issue of how much mobility a person might want to do against what they can do (lack of mobility may only be an issue if one cannot do what they have reason to value). Future studies are needed to validate our framework and to determine if there is a minimum mobility outcome set that can be used for monitoring mobility. Furthermore, apart from life-space mobility, the psychometric properties of ‘actual mobility’ measures have been less well studied. With advancements in technologies for real-world mobility monitoring, large-scale initiatives such as Mobilize-D [15] that examine the use of digital mobility outcomes will be important for generating new knowledge in this space.

In summary, we proposed a unified framework to help advance the science and discourse of mobility measurement for healthy aging. Further studies are needed to validate and test the usability of our framework in aging populations.

## References

[1] World Report on Ageing and Health/World Health Organization . World Health Organization, Editor. Geneva, Switzerland: World Health Organization, 2015.

[2] World Health Organization Team . Decade of Healthy Ageing: Baseline Report. Geneva: World Health Organization. https://www.who.int/publications/i/item/97892400179002021.

[3] Webber SC, Porter MM, Menec VH. Mobility in older adults: a comprehensive framework. Gerontologist 2010; 50: 443–50. 20145017 10.1093/geront/gnq013

[4] Leonardi M, Lee H, Kostanjsek N et al. 20 years of ICF-international classification of functioning, disability and health: uses and applications around the world. Int J Environ Res Public Health 2022; 19: 11321. 10.3390/ijerph191811321. PMC9517056 36141593

[5] Beauchamp MK, Leveille SG, Patel KV et al. What physical attributes underlie self-reported vs. observed ability to walk 400 m in later life? An analysis from the InCHIANTI study. Am J Phys Med Rehabil 2014; 93: 396–404. 24322434 10.1097/PHM.0000000000000034 PMC4304676

[6] Reuben DB, Seeman TE, Keeler E et al. Refining the categorization of physical functional status: the added value of combining self-reported and performance-based measures. J Gerontol A Biol Sci Med Sci 2004; 59: 1056–61. 15528778 10.1093/gerona/59.10.m1056

[7] Guralnik JM, Simonsick EM, Ferrucci L et al. A short physical performance battery assessing lower extremity function: association with self-reported disability and prediction of mortality and nursing home admission. J Gerontol 1994; 49: M85–94. 8126356 10.1093/geronj/49.2.m85

[8] Beauchamp MK, Jette AM, Ward RE et al. Predictive validity and responsiveness of patient-reported and performance-based measures of function in the Boston RISE study. J Gerontol A Biol Sci Med Sci 2015; 70: 616–22. 25512569 10.1093/gerona/glu227 PMC4400398

[9] Beauchamp MK, Bean JF, Ward RE, Kurlinski LA, Latham NK, Jette AM. How should disability be measured in older adults? An analysis from the Boston rehabilitative impairment study of the elderly. J Am Geriatr Soc 2015; 63: 1187–91. 26032351 10.1111/jgs.13453 PMC4478131

[10] Glass TA . Conjugating the ``tenses'' of function: discordance among hypothetical, experimental, and enacted function in older adults. Gerontologist 1998; 38: 101–12. 9499658 10.1093/geront/38.1.101

[11] Veronese N, Honvo G, Amuthavalli Thiyagarajan J et al. Attributes and definitions of locomotor capacity in older people: a World Health Organisation (WHO) locomotor capacity working group meeting report. Aging Clin Exp Res 2022; 34: 481–3. 35133612 10.1007/s40520-022-02080-5 PMC8894172

[12] Beauchamp M, Hao Q, Kuspinar A, et al. Measures of perceived mobility in community-dwelling older adults: A systematic review of psychometric properties. Age and Ageing 2023; afad124. 10.1093/ageing/afad124. PMC10615037 37902516

[14] Kuspinar A, Mehdipour A, Beauchamp MK, et al. Assessing the measurement properties of life-space mobility measures in community-dwelling older adults: a systematic review. Age and Ageing 2023; afad119. 10.1093/ageing/afad119. PMC10615067 37902523

[13] Honvo G, Sabico S, Veronese N, et al. Measures of attributes of locomotor capacity in older people: a systematic literature review following the COSMIN methodology. Age and Ageing 2023; afad139. 10.1093/ageing/afad139. PMC10615073 37902521

[15] Mikolaizak AS, Rochester L, Maetzler W et al. Connecting real-world digital mobility assessment to clinical outcomes for regulatory and clinical endorsement-the mobilise-D study protocol. PLoS One 2022; 17: e0269615. 10.1371/journal.pone.0269615. 36201476 PMC9536536

